# Ferromagnetic Resonance Revised – Electrodynamic Approach

**DOI:** 10.1038/s41598-017-05827-7

**Published:** 2017-07-18

**Authors:** Jerzy Krupka, Pavlo Aleshkevych, Bartlomiej Salski, Pawel Kopyt, Adam Pacewicz

**Affiliations:** 1 Institute of Microelectronics and Optoelectronics, Warsaw University of Technology, Warsaw, 00662 Poland; 2 Institute of Physics, Polish Academy of Sciences, Warsaw, 02668 Poland; 3 Institute of Radioelectronics and Multimedia Technology, Warsaw University of Technology, Warsaw, 00665 Poland

## Abstract

Resonance in a ferromagnetic sphere, known in the body of literature as the mode of uniform precession, has recently been proven to be magnetic plasmon resonance (MPR). This finding has prompted research which is presented in this paper on the relation between the Q-factor at the MPR and the ferromagnetic resonance (FMR) linewidth Δ*H*, which is a parameter of magnetized gyromagnetic materials. It is proven in this paper that Δ*H* can be unequivocally determined from the Q-factor measured at the MPR, if all losses in the resonance system are properly accounted for. It can be undertaken through a rigorous but simple electrodynamic study involving the transcendental equation, as proposed in this paper. The present study also reveals that electric losses have a substantially reduced impact on Δ*H* due to the large magnetic to electric energy storage ratio at the MPR. Theoretical results are supported by measurements of the Q-factors on a monocrystalline yttrium iron garnet (YIG) sphere.

## Introduction

Monocrystalline YIG is a gyromagnetic material with the lowest known magnetic loss and it has been used for decades in microwave filters and oscillators as well as in spintronics. Theories of the FMR, spin waves, and modes of operation of YIG resonators were developed over 60 years ago^1–12^ but YIG is still one of the most frequently studied ferrimagnetics^13–18^. Only recently it has been shown^18^ that for spherical samples the most pronounced resonance mode that is responsible for the operation of YIG-based devices, often called the mode of uniform precession, does not correspond to the FMR frequency where the YIG has the largest magnetic absorption, but to the first MPR where the effective permeability *μ*_*r*_ of the clockwise circularly polarized (CCP) mode is approaching the value of −2 (*μ*_*r*_ ≈ −2).

The electric and magnetic plasmon phenomena have been studied for a long time by researchers working on metamaterials^19–26^. It has been shown that metallic split-ring resonators as well as U-shaped nanostructures can exhibit negative effective permeability at resonances^19–26^ occurring from microwaves to the optical spectral range, depending on the sizes of those structures. Such metamaterials usually have relatively large magnetic losses due to the finite conductivity of applied metals. Monocrystalline YIG seems to be an alternative to metals in the construction of low loss metamaterials at microwaves and millimeter waves, as it exhibits negative *μ*_*r*_ and low magnetic losses at the MPR (measured Q-factors of YIG resonators are at the order of a few thousand). From a viewpoint of the aforementioned new findings related to the plasmonic nature of resonances occurring in YIG spheres^18^, which were confirmed both theoretically and experimentally, another emerging issue has to be thoroughly addressed. Namely, the origin of losses contributing to the Q-factor occurring at the MPR and its relation to the FMR linewidth ΔH, which is a commonly used parameter of ferromagnetic materials. For that purpose, rigorous analytic electrodynamic modeling with the aid of a properly defined transcendental equation, accounting for a dispersive characteristic of gyromagnetic materials, was applied in order to investigate this issue and is described in the present paper.

The FMR phenomenon is quantitatively described with a permeability tensor derived from the Landau-Lifshitz-Gilbert equations. If a uniform static magnetic bias is applied along the principal axis, diagonal and off-diagonal relative components of the permeability tensor take the following form^11, 18^:

1 $$\mu =1+\frac{{H}_{0r}+j\alpha \hat{w}}{{H}_{0r}^{2}-{\hat{w}}^{2}+2\,j\alpha {H}_{0r}\hat{w}}$$

2 $$\kappa =\frac{\hat{w}}{{H}_{0r}^{2}-{\hat{w}}^{2}+2j\alpha {H}_{0r}\hat{w}}$$

where: *H*_0*r*_ = *H*_0_/*M*_*S*_, $\hat{w}\,=\hat{f}/{f}_{m}$, *f*_*m*_ = *γM*_*S*_, *H*_0_ is the static magnetic bias inside the sample, *M*_*S*_ is the saturation magnetization, *γ* = 35.19 MHz/(kA/m), *α* is a Gilbert damping factor, and $\hat{f}$ is the complex frequency.

Experiments show that *α* varies with frequency, while the relaxation time, *τ* = 1/(*αγH*_0_), and the FMR linewidth, ${\rm{\Delta }}H=2\alpha {H}_{0}=2/(\gamma \tau )$, do not. The internal bias *H*_0_ is related to the external bias *H*_*e*_ by the formula ${H}_{0}={H}_{e}-\frac{{{\rm{M}}}_{{\rm{S}}}}{3}-\delta {H}_{a}$. The effective anisotropy field *δH*_*a*_ depends on the orientation of the YIG crystal with respect to *H*_0_ and can vary from ca. −4.6 kA/m for easy orientation to +6.9 kA/m for hard orientation by rotating the sample around its [110] crystallographic axis.

If the microwave magnetic field of the CCP mode is transverse to *H*_0_, the effective permeability tensor becomes diagonal with the following complex scalar relative quantity:

3 $${\mu }_{r}=\mu +\kappa ={{\mu }_{r}}^{\prime}-j{{\mu }_{r}}^{\prime\prime}$$

Equation 3 exhibits resonance properties in the same way as Equations (1) and (2). The internal static magnetic bias is equal to ${H}_{0,FMR}=\frac{f}{\gamma }$ and ${H}_{0,MPR}=\frac{f}{\gamma }-\frac{1}{3}{M}_{S}$ at the FMR and the MPR, respectively. It has been shown^18^ that the real part of the scalar permeability is ${{\mu }_{r}}^{{\prime} }=-2$ at the MPR for the infinitesimally small ferromagnetic sphere. Consequently, the MPR appears at ${H}_{e,MPR}=\frac{f}{\gamma }$ provided that *δH*_*a*_ = 0. Although this formula appears to be similar to the formula used to describe the FMR, it is given for the external magnetic bias in the free space and not inside the gyromagnetic medium. As will be shown in this paper, the MPR is practically observed in the vicinity of ${{\mu }_{r}}^{{\prime} }=-2$, due to the finite size of the sample and the metal enclosure. Further, it is noteworthy that although the magnetostatic model (MS) theory^3^ predicts the location of the mode of uniform precession at ${{\mu }_{r}}^{{\prime} }=-2$, it does not allow the determining of its Q-factor at microwave frequencies.

**Figure 1:**
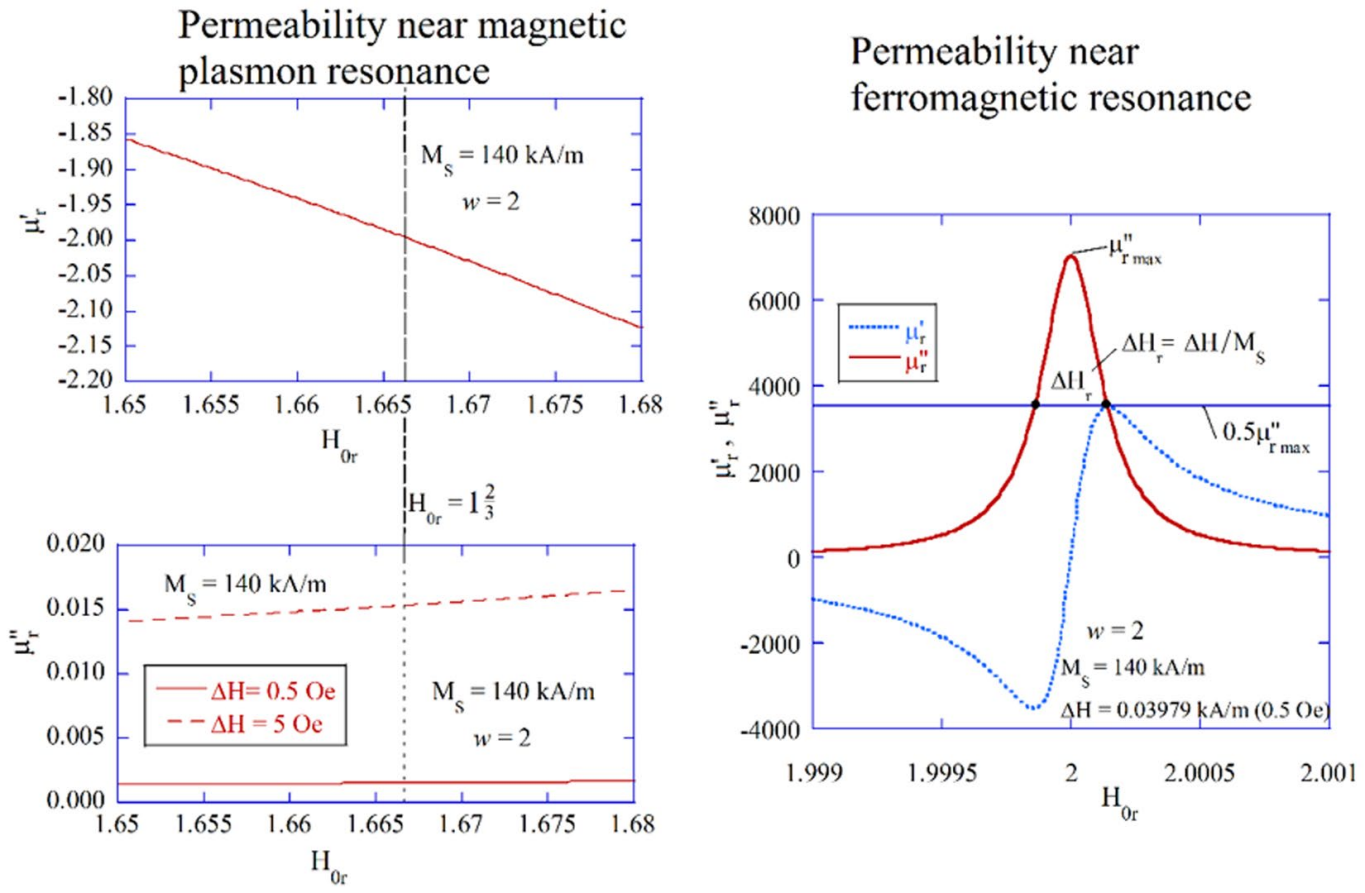
Permeability. The real and imaginary components of the complex scalar relative permeability *μ*_*r*_ = *μ* + *κ* in the vicinity of the MPR (on the left) and FMR (on the right).

Let us clarify the relationship between *Q* at the MPR, which is commonly measured, and the FMR linewidth *ΔH*, which is usually sought. The dependence of the complex scalar permeability components on H_0r_ in the vicinity of the FMR and the MPR are presented in Fig. 1. The imaginary part of the permeability is at a maximum at the FMR, which is ${{\mu }^{\prime\prime}}_{r,FMR}={{\rm{H}}}_{0}/{\rm{\Delta }}H$, and it decreases to ${{\mu}^{\prime\prime}}_{r,MPR}=9\frac{{\rm{\Delta }}H}{2{H}_{0}}w$ at the MPR. This latter expression is derived from Equations (1–3) by assuming the MPR condition (*H*_0r_ = *w* − 1/3). It is essential to note that ${{\mu }^{{\prime\prime} }}_{r,MPR}$ decreases with ΔH, which is opposite to ${{\mu }^{{\prime\prime}}}_{r,FMR}$. As is shown in Fig. 1, for a typical YIG sphere with ΔH = 0.5 Oe, ${{\mu }^{{\prime\prime}}}_{r,MPR}$ is more than 6 orders of magnitude smaller then ${{\mu}^{{\prime\prime}}}_{r,FMR}$ and does not exhibit resonance properties. Employing the MS field approximation and perturbation theory (PT) to a cavity containing a small gyromagnetic sphere, one can predict the MPR at ${{\mu }_{r}}^{{\prime}}=-2$. However, the Q-factor values obtained utilizing PT theory do not agree with experiments with narrow linewidth samples with ΔH < 10 Oe. According to PT, the Q-factor of the cavity should monotonically decrease with the linewidth ΔH, while in experiments the opposite phenomenon takes place for the narrow linewidth samples. The Q-factors observed experimentally for YIG samples are at the order of a few thousand and they increase with decreasing linewidth ΔH. Such behavior can be explained using an electrodynamic approach for dispersive media as is presented in the next section.

### Quality factor in dispersive magnetodielectric media

The solution of the eigenvalue problem of free oscillations of a resonance system surrounded by a perfectly conducting surface is in the form of complex eigenfrequencies ${\hat{\omega }}_{s}={\omega }_{s}^{{\prime}}+j{\omega }_{s}^{{\prime\prime}}$, corresponding to specific modes with the Q-factors that, by definition, can be computed as:

4 $${Q}_{s}=\frac{{\omega }_{s}^{{\prime}}}{2{\omega }_{s}^{\prime\prime}}$$

As shown in ref. 27, the *Q*–factor of the *s*-th mode of free oscillations of the resonator containing dispersive media can be expressed as:

5 $${Q}_{s}=\frac{\frac{1}{4}\{{\iiint }_{V}\frac{\partial [({\omega }_{s}^{{\prime} }\,{\varepsilon }_{a}^{{\prime} }({\omega }_{s}^{{\prime} })]}{\partial \omega }{|{{\boldsymbol{E}}}_{{\boldsymbol{s}}}|}^{2}dv+{\iiint }_{V}\frac{\partial [{\omega }_{s}^{{\prime} }\,{\mu }_{a}^{{\prime} }({\omega }_{s}^{{\prime} })]}{\partial \omega }{|{{\boldsymbol{H}}}_{{\boldsymbol{s}}}|}^{2}dv\}}{\frac{1}{2}({\iiint }_{V}{\varepsilon }_{a}^{{\prime\prime} }({\omega }_{s}^{{\prime} }){|{{\boldsymbol{E}}}_{{\boldsymbol{s}}}|}^{2}dv+{\mu }_{a}^{{\prime\prime} }({\omega }_{s}^{\text{'}}){\iiint }_{V}{|{{\boldsymbol{H}}}_{{\boldsymbol{s}}}|}^{2}dv)}$$

where ${\varepsilon }_{a}={\varepsilon }_{a}^{{\prime} }-j{\varepsilon }_{a}^{{\prime\prime} }$ (${\mu }_{a}={\mu }_{a}^{{\prime} }-j{\mu }_{a}^{{\prime\prime} }$) is the absolute complex permittivity (permeability) of the considered media.

The denominator in Equation (5) denotes the average total energy dissipated in the resonator (*P*_*loss*_), while the numerator can be interpreted as the average total electromagnetic (EM) energy stored in the resonator.

A similar expression for the total EM energy stored in dispersive media can be found in the body of literature^28–32^. For nondispersive media $\frac{\partial [({\omega }_{s}^{{\prime} }\,{\varepsilon }_{a}^{{\prime} }({\omega }_{s}^{{\prime} })]}{\partial \omega }={\varepsilon }_{a}^{{\prime} }$ and $\frac{\partial [{\omega }_{s}^{{\prime} }\,{\mu }_{a}^{{\prime} }({\omega }_{s}^{{\prime} })]}{\partial \omega }={\mu }_{a}^{{\prime} }$, so that Equation (5) reduces to the well-known formula:

6 $${Q}_{s}=\frac{\frac{1}{2}\{{\iiint }_{V}{w}_{e}dv+{\iiint }_{V}{w}_{m}dv\}}{{P}_{loss}}$$

where ${w}_{e}=\frac{1}{4}{\varepsilon }_{a}^{{\prime} }{|{{\boldsymbol{E}}}_{{\boldsymbol{s}}}|}^{2}$ and ${w}_{m}=\frac{1}{4}{\mu }_{a}^{{\prime} }{|{{\boldsymbol{H}}}_{{\boldsymbol{s}}}|}^{2}$ represent, respectively, the average electric and magnetic energy densities stored in the resonator. Eventually, one can obtain the following formula:

7 $${Q}_{s}=\frac{{W}_{T}}{{W}_{loss}}=\frac{({W}_{E}+{W}_{M})}{{W}_{loss}}=\frac{{\omega }_{s}^{{\prime} }({W}_{E}+{W}_{M})}{{P}_{loss}}$$

where *W*_*T*_, *W*_*E*_, and *W*_*M*_ are the average total, electric and magnetic energies stored in the resonator, respectively. Usually, it can also be assumed that the following condition is met at resonance *W*_*E*_ = *W*_*M*_, which leads to:

8 $${Q}_{s}=\frac{2{\omega }_{s}^{{\prime} }{W}_{M}}{{P}_{loss}}=\frac{2{\omega }_{s}^{{\prime} }{W}_{E}}{{P}_{loss}}$$

However, as it has already been pointed out elsewhere in the body of literature^28, 29^, *Q*_*s*_ cannot be determined correctly using Equation (8) within the framework of phenomenological electrodynamics if media are dispersive. Nevertheless, it is postulated in this paper that Equation (5) can still be used to determine *Q*_*s*_ if the assumption that *W*_*E*_ = *W*_*M*_ is not imposed.

### Quality factor in spherical ferromagnetic samples

**Figure 2:**
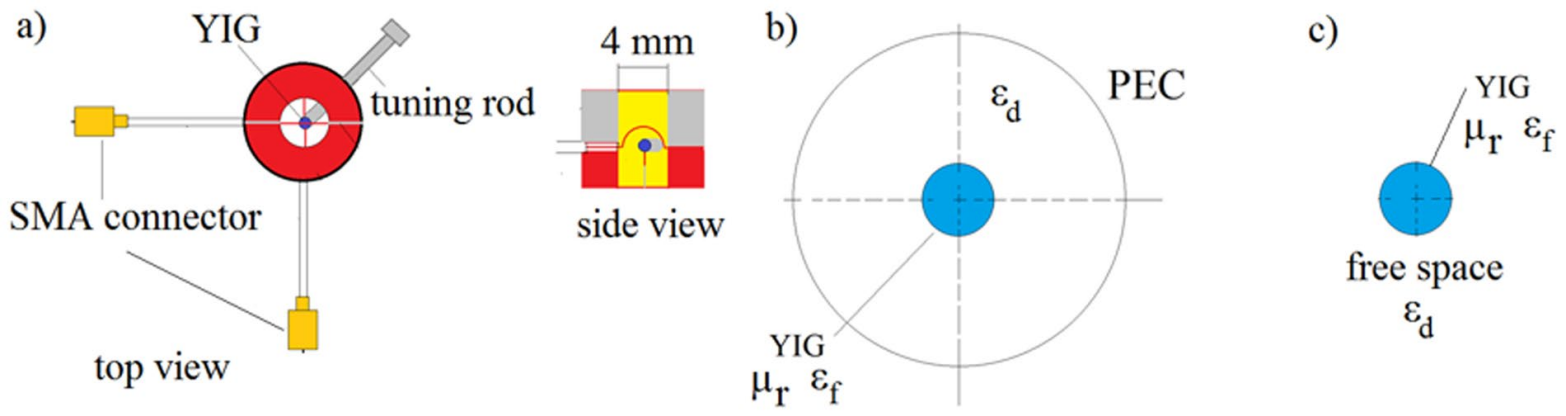
Setup. (**a**) Experimental setup. Model of a gyromagnetic sphere (**b**) in the conducting enclosure and (**c**) in the free space used in theoretical simulations.

In this section, numerical analysis of the Q-factor of free oscillations for the dominant MPR mode is undertaken. The experimental setup is shown in Fig. 2a, while the corresponding simplified models used in the theoretical analysis are depicted in Fig. 2b,c. The YIG sphere of radius *R*_*1*_ ≈ 0.25 mm was mounted on a beryllia ceramic rod with [110] crystallographic orientation with respect to the rod’s axis. It was shown in our earlier paper^18^ that the microwave magnetic field at the MPR is orthogonal to *H*_0_.

Thus, CCP plasmonic modes can be rigorously analyzed in an appropriately chosen spherical coordinate system rotating synchronously with the rotating EM fields, assuming that permeability *μ*_*r*_ is a complex scalar dispersive quantity. The CCP MPR corresponds in that coordinate system to the non-rotating TE_101_ mode, where the subscripts indicate elevation, azimuthal, and radial mode indices, respectively. The transcendental equation (TDE) for the TE_n0p_ mode in a gyromagnetic sphere is given as follows^18^:

9 $$\begin{array}{cc}\{n{J}_{n+\frac{1}{2}}(k{R}_{1})-k{J}_{n-\frac{1}{2}}(k{R}_{1})\}{H}_{n+\frac{1}{2}}^{(2)}({k}_{0}{R}_{1}) & -{\mu }_{r}\{n{H}_{n+\frac{1}{2}}^{(2)}({k}_{0}{R}_{1})-{k}_{0}{H}_{n-\frac{1}{2}}^{(2)}({k}_{0}{R}_{1})\}\\  & \times {J}_{n+\frac{1}{2}}(k{R}_{1})=0\end{array}$$

where $k=\hat{\omega }/c{({\varepsilon }_{f}{\mu }_{r})}^{0.5}$, ${k}_{0}=\hat{\omega }/c{({\varepsilon }_{d})}^{0.5}$, *ε*_*f*_ (*ε*_*d*_) is the relative complex permittivity of the sphere (medium surrounding the sphere), *μ*_*r*_ is the relative complex permeability of the sphere as given in Equation (3), *n, p* are elevation and radial mode indices, respectively, *c* is the speed of the EM wave in a vacuum, *J* (*H*) are Bessel (Hankel) functions.

It is noteworthy that material properties and frequency should be complex, both in Maxwell curl equations and in Equation (9). Consequently, if the gyromagnetic sphere is located in the free space, where radiation losses may occur, the solution ${\hat{\omega }}_{s}$ of Equation (9) is complex (*s* denotes a unique pair of subscripts *n* and *p*) even if materials are lossless. Formally, it results from the properties of the Hankel function (complex valued for real arguments). The Q-factor of free oscillating modes can be calculated in two ways, which should be consistent with each other. The first method, as given in Equation (4), relies directly on the solution of Equation (9) with respect to ${\hat{\omega }}_{s}$, while the other method is based on Equation (5), which requires calculating and properly integrating EM fields at ${\hat{\omega }}_{s}$. If the sphere is situated at the center of a perfectly conducting spherical cavity, the TDE needs to be modified^18^. In that case, eigenfrequencies ${\hat{\omega }}_{s}$ are real for lossless media as radiation losses are absent. All the computations presented in this paper were performed by employing in-house developed codes that are available to interested parties for their own studies^33^.

**Figure 3:**
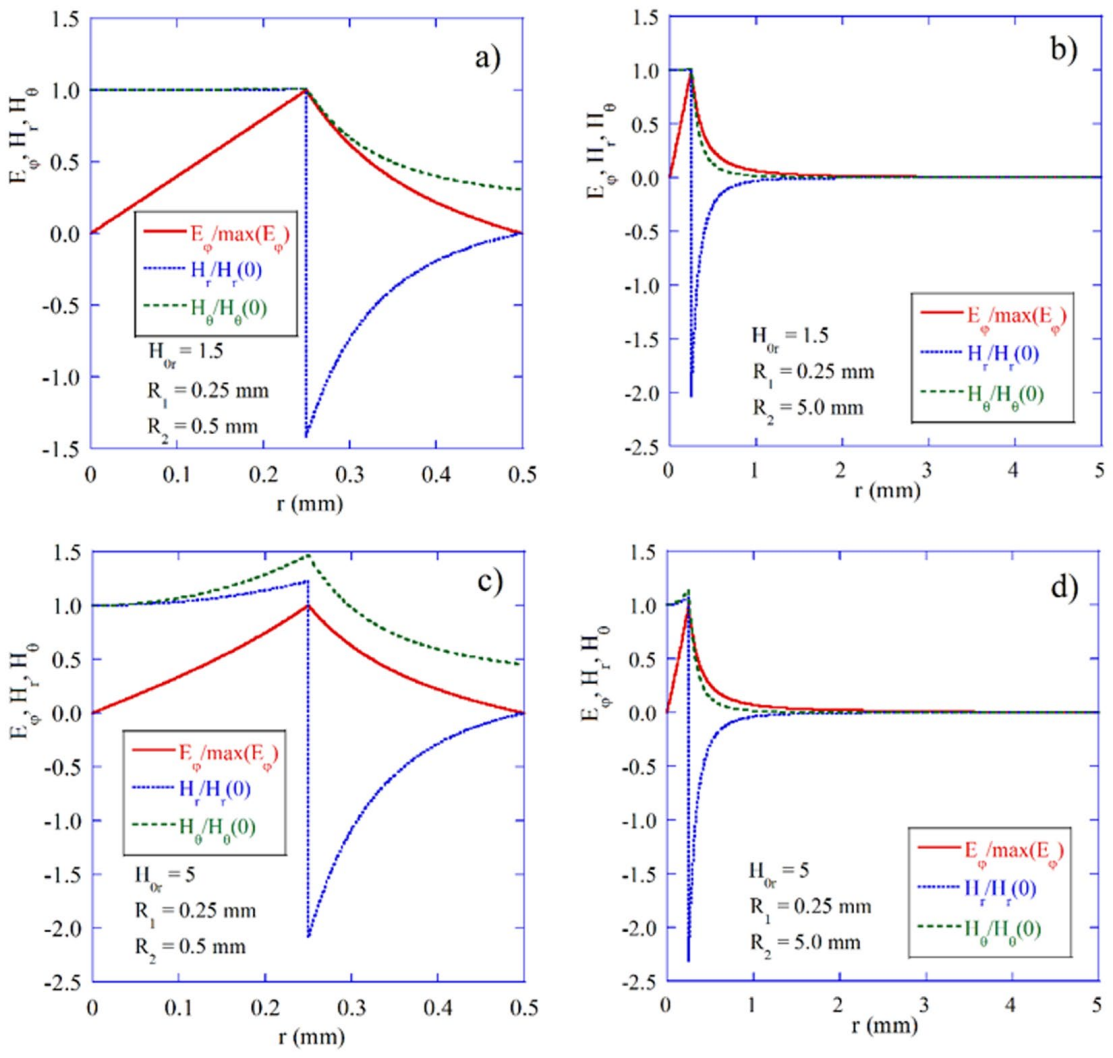
EM fields. Distribution of EM fields at the MPR for the gyromagnetic sphere situated at the center of spherical cavities of two different sizes calculated for two magnetic field bias levels.

Computations were performed for the following parameters: *M*_*S*_ = 140 kA/m, *δH*_0_ = 0, ${\varepsilon }_{f}^{{\prime} }=16$, ${\rm{\Delta }}H=0.5$ Oe, *R*_1_ = 0.25 mm. Figure 3 shows the distribution of EM fields at the MPR for the gyromagnetic sphere situated at the center of a perfectly conducting spherical cavity of two different sizes, *R*_2_ = 0.5 mm and *R*_2_ = 5 mm, and for two different magnetic biases, H_0r_ = 1.5 and H_0r_ = 5. It was noted that at low bias the microwave magnetic field inside the sphere is almost uniform, while for stronger bias it becomes more concentrated at the sphere’s surface. However, in both cases EM fields are evanescent outside the sphere, although they decay faster at lower bias. It was noted that even for large metal enclosures EM energy is concentrated in the vicinity of the sphere’s surface. EM fields are very similar if the sphere is situated in the free space, hence these are not presented here.

**Figure 4:**
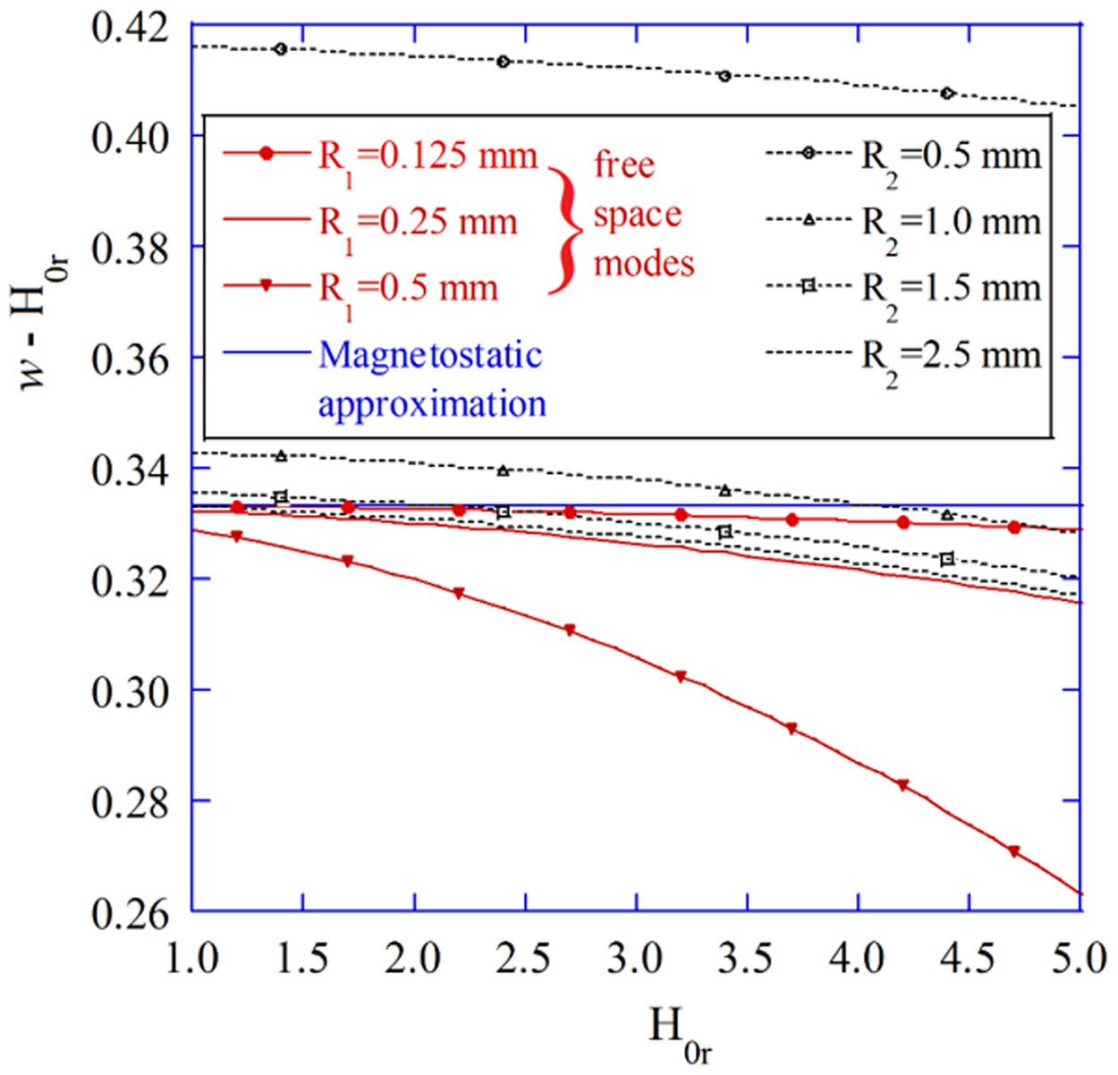
MPR frequency variation. Variation of the MPR frequency with the radius of the sample (*R*_1_) and the radius of the perfectly conducting spherical enclosure (*R*_2_) as computed with the TDE. Influence of the conducting enclosure (curves in black color) is shown only for *R*_1_ = 0.25 mm.

The reduction of the sphere’s radius to 0.125 mm, for example, can improve field uniformity inside, but the realization of such small samples having decent surface quality is hardly feasible. The influence of the shape quality is manifested as the deviation of the MPR frequency which is especially visible at higher frequencies. Figure 4 shows the difference (*w* − *H*_0*r*_) as a function of *H*_0*r*_ for 3 radii of the sphere in the free space and for the sphere having radius *R*_1_ = 0.25 mm, situated in 4 different and perfectly conducting enclosures. It can be seen that the MS condition (*w* = *H*_0*r*_ + 1/3) is nearly met only at low bias (*H*_0*r*_ = 1) and for *R*_1_ ≤ 0.25 mm. For larger spheres, differences between electrodynamic and magnetostatic results become significant. That case is usually accounted for approximately^6^ by assuming that the effective gyromagnetic ratio (g-factor) varies, which is not correct. Similar behavior with the impact of *H*_0_ on the MPR frequency can be observed for the sphere in a perfectly conducting spherical cavity. It is also seen that for sufficiently large enclosures (*R*_2_ ≥ 2.5 mm), results of (*w* − *H*_0*r*_) computations for a shielded sphere converge to the results of *w* − *H*_0*r*_ for the sphere in the free space.

It is noteworthy that the deviation of the MPR frequency from the MS condition results in the change of the mode’s effective permeability. For increasing *H*_0_, when *w* − *H*_0*r*_ < 1/3, permeability decreases below -2, while the placement of the sphere in the finite size metal enclosure, when *w* − *H*_0*r*_ > 1/3, results in the increase of permeability above -2. For instance, if the shield is two-times larger than the gyromagnetic sphere (see the curve for *R*_*2*_ = 0.5 mm in Fig. 4), the effective permeability at the MPR is *μ*_*r*_ ≈ −1.4 for *w* − *H*_0*r*_ < 0.415, while in the case of the sphere with *R*_1_ = 0.5 mm located in the free space, *μ*_*r*_ ≈ −2.8 for *w* − *H*_0*r*_ < 0.265. These *μ*_*r*_ values were estimated using Equations (1)–(3).

**Figure 5:**
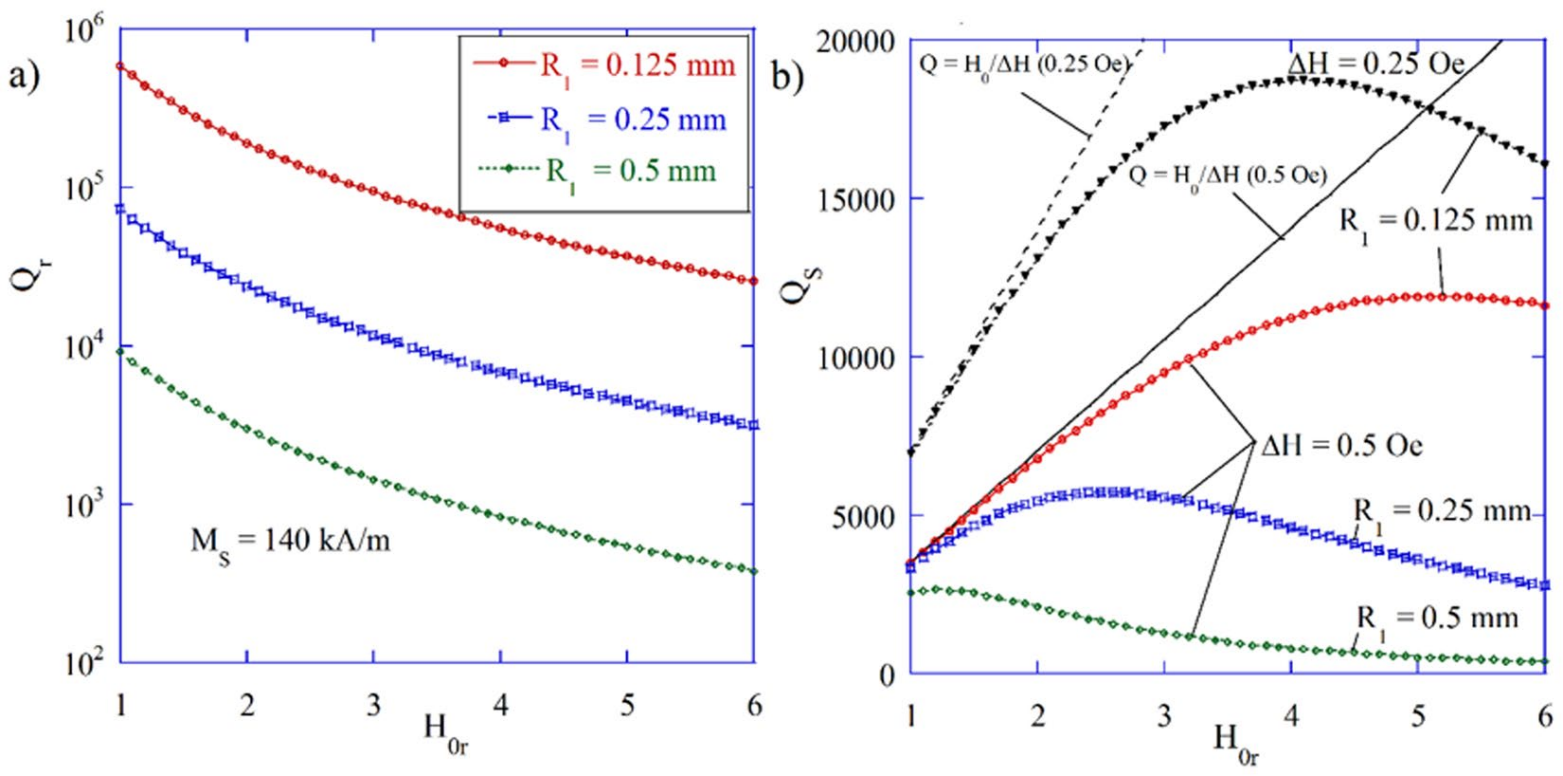
Q-factor at the MPR for the gyromagnetic sphere in the free space. The Q-factor of the gyromagnetic spheres of different radii situated in the free space, as computed with the TDE, (**a**) with radiation losses only (*ΔH* = 0) and (**b**) with both radiation and magnetic losses (*ΔH* > 0).

Figure 5a shows the Q-factor versus *H*_*0r*_ for the lossless gyromagnetic sphere (*ΔH* = 0) in the free space, so that only radiation losses are present. It can be seen that *Q* significantly depends on the radius of the sample, but it can be *Q* > 10^5^ for *R*_1_ = 0.125 mm, provided that *H*_0*r*_ < 3. This is 4 decades larger than in the case of a TE_101_ mode in a similar spherical dielectric resonator (*ε*_*r*_ = 16)^34^. However, the Q-factor due to radiation losses can be as low as ca. 300 for *H*_0*r*_ = 6 and *R*_1_ = 0.5 mm (see Fig. 5a). It indicates that the estimation of radiation losses of gyromagnetic spheres is important for their practical applications in unshielded or partly-shielded structures, especially at millimeter wave frequencies, where they can be larger than the magnetic losses. According to Fig. 5b, for typical samples having ${\rm{\Delta }}H=0.5$ Oe and *R*_1_ = 0.25 mm, the maximum of the unloaded *Q* is ca. 6000 at *H*_0*r*_ ≈ 2.5, which agrees well with experimental results available in the body of literature^7, 9^. It is noteworthy that, theoretically, the *Q*-factor of shielded samples linearly increases with *H*_*0*_, as has been confirmed numerically^26^. Simultaneously, however, conduction losses in the metal enclosure increase with frequency, which practically limits the maximum *Q* that can be obtained by employing spherical ferromagnetic resonators (see Fig. 5b).

For low magnetic bias (*H*_0*r*_ < 2), when radiation losses are small, *Q* converges to the asymptotic limit ${H}_{0}/{\rm{\Delta }}H$ so that ${\rm{\Delta }}H={H}_{0}/{Q}_{S}$. It leads to an essential conclusion that a few conditions, such as the small size of the sample, low magnetic bias, and weak coupling, have to be satisfied to determine Δ*H* from the measured *Q*_*S*_.

Another issue that is addressed in this paper, which reveals unusual properties of ferromagnetic spheres, is the influence of dielectric losses on the total *Q* at the MPR. Consider the sphere without magnetic losses (*ΔH* = 0) having *R*_1_ = 0.25 mm located in a perfectly conducting spherical cavity with *R*_2_ = 2.5 mm to avoid, for simplicity, radiation and conduction losses. If the dielectric loss tangent is assumed to be the same inside and outside of the ferromagnetic sphere (*tanδ* = 10^−4^), *Q* computed with Equation (4) is in the range from 6.16 × 10^6^ at *H*_0*r*_ = 1.0 down to 1.44 × 10^6^ at *H*_0*r*_ = 5. The same result can be obtained by employing the so-called incremental frequency rules for computing electric energy filling factors, ${p}_{ei}=2|\frac{\partial {\omega }_{s}^{{\prime} }}{\partial {\varepsilon }_{i}^{{\prime} }}|\frac{{\varepsilon }_{i}^{{\prime} }}{{\omega }_{s}^{{\prime} }}$, where *i* denotes permittivity of the sphere (f) or dielectric (d) surrounding of the sphere. The incremental frequency rules are valid, if the resonance frequency is an analytic function of permittivity, which is satisfied for Equation (9).

It should be noted, however, that if Equation (8) was used instead of Equation (7), by assuming that *W*_*M*_ = *W*_*E*_ at the MPR, *Q* would be as small as 10^4^ which corresponds well to 1/*tanδ*. Such a relation between *Q* and *tanδ* is commonly expected for a nondispersive dielectric resonator surrounded by the medium with the same *tanδ* and with other losses neglected. The only reasonable explanation for larger than typically expected Q-factors due to dielectric losses is that *W*_*M*_ is more than 2 decades larger than *W*_*E*_. Two additional measures were undertaken to prove that such an inequality does indeed take place. First, *Q* was calculated using Equation (5) by numerical integration of EM fields and the results agree to within 1–2% with those obtained using Equations (4) and (9). The second proof for the large disproportion between *W*_*M*_ and *W*_*E*_ is experimental. For that purpose, measurements of the unloaded *Q* of the YIG sphere located in the filter structure open to the free space, as shown in Fig. 2a, were performed. Subsequently, the filter was inserted into a glass container filled with electrically lossy propanol^35^, which is one of the standard reference liquids with permittivity described by a Debye model^35^. Permittivity of propanol exhibits dispersion at microwave frequencies and its value at 6 GHz is *ε*_*d*_ ≈ 3.56 − *j*1.18.

**Figure 6:**
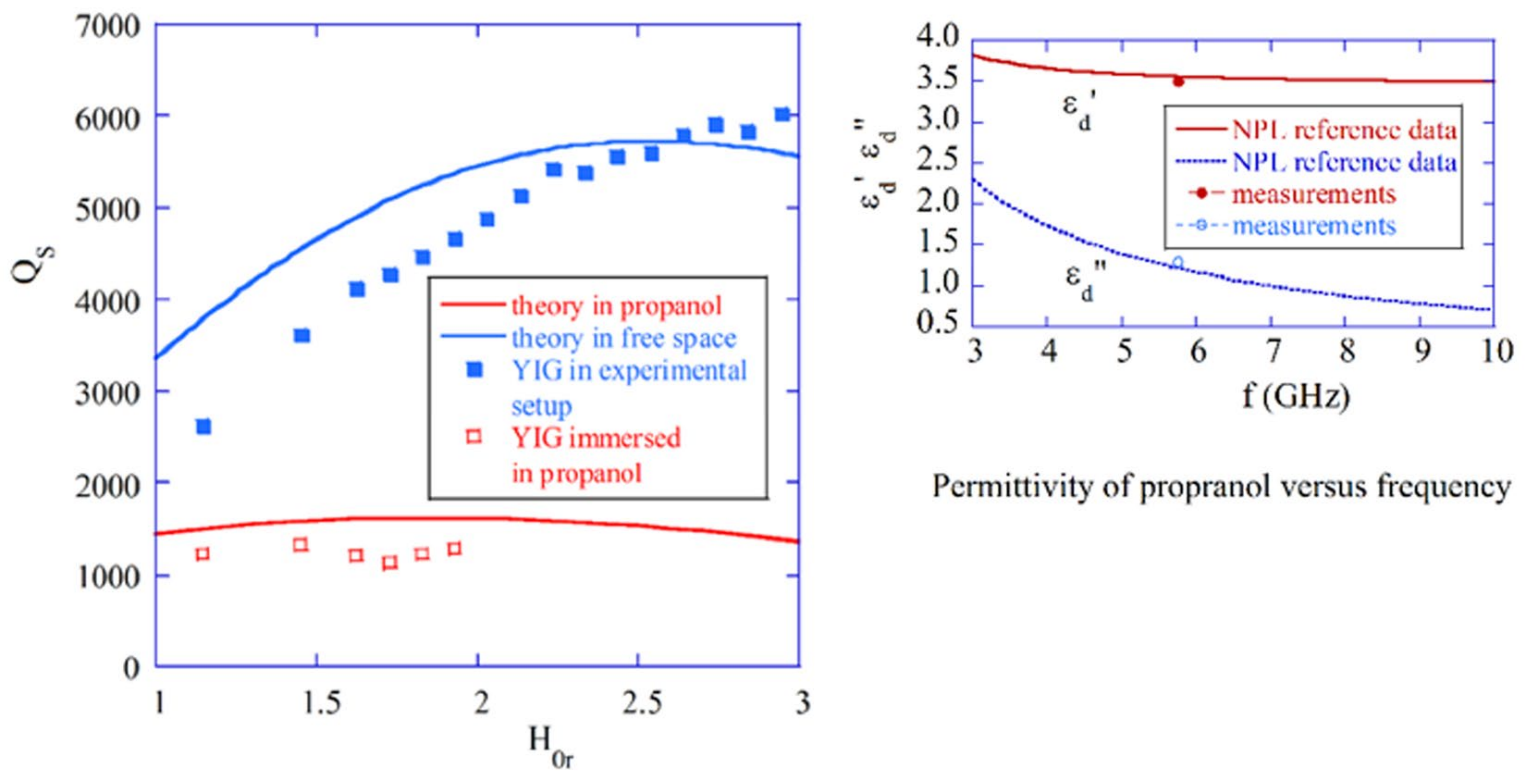
YIG sphere in the free space and immersed in propanol. Computed and measured Q-factors due to the magnetic, dielectric and radiation losses versus *H*_*0r*_ for the YIG sphere in the free space and in propanol. Computations were performed for *R*_1_ = 0.25 mm, ${\rm{\Delta }}H=0.5\,$Oe, *M*_*S*_ = 140 kA/m and propanol permittivity described by a Debye model^35^.

Q-factors numerically computed with Equation (5) for the YIG sphere in the free space and surrounded by propanol are shown in Fig. 6, together with the corresponding Q-factors, measured with the Agilent Technologies PNA-X vector network analyzer. The experimental setup with the YIG sample was placed between the poles of a magnet in the Bruker EPR spectrometer. In numerical computations the linewidth value was assumed to be ${\rm{\Delta }}H=0.5$ Oe, but experiments show that it is about 20% larger for the measured YIG sample. As can be seen in Fig. 6, in the case of the free space surrounding, *Q* increases with *H*_*0*_ until radiation losses prevail so that *Q* starts to decrease in the theoretical model. For *H*_0*r*_ > 2.5, the measured Q-factors are then larger than the theoretical factors since the YIG sphere is partly shielded in the experimental setup by the mounting fixture of the coupling loops, so that the radiation losses are smaller than for the free space analytical model.

According to Fig. 6, despite the immersion of the YIG sphere in lossy propanol, *Q* is still over 1000, which agrees with the theoretical results. As the measurements were not reliable at *H*_0*r*_ > 2, where coupling was too weak for accurate 3 dB points measurements, these results are omitted in Fig. 6. As the electric energy filling factor in propanol is about *p*_*ed*_ = 20% and its dielectric loss tangent is about *tanδ*_*d*_ = 1.18/3.56 ≅ 0.3, *Q* should drop to about 15, if *W*_*M*_ = *W*_*E*_. However, the measured value was *Q* > 1000, which confirms that ${W}_{M}\gg {W}_{E}$ at the MPR. Detailed computations show that the *W*_*M*_/*W*_*E*_ ratio slightly decreases with frequency. For the completeness of the analysis, the contribution of conduction losses of metal shield was also addressed by employing the incremental frequency rule. It turned out that if the shield is made of metal that is a good conductor, such as silver or copper, its contribution to the total *Q* can be neglected for R_2_ > 5R_1_. This is rather expected as the tangential magnetic field component decays exponentially with the radius, which is seen in Fig. 3.

## Discussion and Conclusions

Rigorous analytic electrodynamic model of the MPR in the ferromagnetic sphere containing dispersive and lossy media allows explaining the origin of losses and determining the corresponding Q-factors of spherical resonators operating at the MPR. It has been shown that most of the measurements of the ferromagnetic linewidth ΔH of YIG spheres are in fact undertaken at the MPR, and not at the FMR (as usually expected). That finding should be emphasized as many researchers still consider the mode of uniform precession simply as a FMR mode, where the magnetic losses are at a maximum. On the contrary, magnetic losses are relatively small at the MPR frequency as the Q-factor of YIG resonators is of the order of 5000. If a small YIG sample is placed into a cavity and MPR conditions are satisfied, EM energy is stored mostly in the YIG sphere, and the measured Q-factor is at the same order as for the YIG sphere in the free space. This means that, depending on the initial value, the total Q-factor of the cavity either decreases or increases, reaching the Q-factor of the YIG sphere at the MPR. Perturbation theory fails in such cases as it erroneously implies that the Q-factor always decreases with ΔH.

A large disproportion between the average magnetic and the average electric energy stored in the resonance structure has been predicted using the proposed TDE and has also been confirmed experimentally. That property was demonstrated by immersing the YIG sphere in lossy liquid, the electric losses of which had a much smaller impact on the total Q-factor than might be expected if *W*_*M*_ = *W*_*E*_. It has also been shown that nonlinearity in a ferromagnetic tuning characteristic at high frequencies is not associated with the changes of the effective g-factor but with the influence of a metal shield and the size of the sample on the MPR condition. As the electrodynamic model presented in this paper is rigorous and simple, it should replace the approximate magnetostatic model that has been used in the analysis of various devices containing ferromagnetic spheres for the last 60 years. Contrary to the MS model, the electrodynamic model allows to rigorously determine the Q-factors of spherical ferromagnetic resonators (including radiation losses), as well as the influence of the size of the YIG sphere and metal enclosure on the MPR conditions (see Fig. 4). It is noteworthy that the presented measurement setup can be used for accurate linewidth measurements of narrow linewidth ferromagnetic materials. The ASTM standard^36^, which specifies the methods of the ferromagnetic linewidth measurements, is based on perturbation theory and as such can be applied only for materials with ΔH > 10 Oe. The method that employs the measurement setup proposed in this paper would be complementary to the ASTM Standard, allowing the linewidth measurements of materials having ΔH < 10 Oe with practically no limit for the lowest measurable linewidth value.

The size of spherical YIG samples that are considered in this paper, with diameters ranging from 0.25 mm to 1 mm, corresponds to typical dimensions of commercially available samples. Those interested parties with respect to the analysis of ferromagnetic resonators having different size, different saturation magnetization, and/or different linewidth may use our freeware MATLAB programs^33^ for this purpose.
